# Extraction of Attenuation and Backscattering Coefficient along Hollow-Core Fiber Length Using Two-Way Optical Time Domain Backscattering

**DOI:** 10.1021/acsphotonics.4c00859

**Published:** 2024-09-24

**Authors:** Xuhao Wei, Eric Numkam Fokoua, Francesco Poletti, Radan Slavík

**Affiliations:** † Optoelectronics Research Centre, University of Southampton, Southampton SO17 1BJ, U.K.; ‡ Microsoft U.K., Romsey SO51 9DL, U.K.

**Keywords:** fiber optics, characterization of hollow-core fiber, backscattering coefficient, distributed loss, optical time domain reflectometry

## Abstract

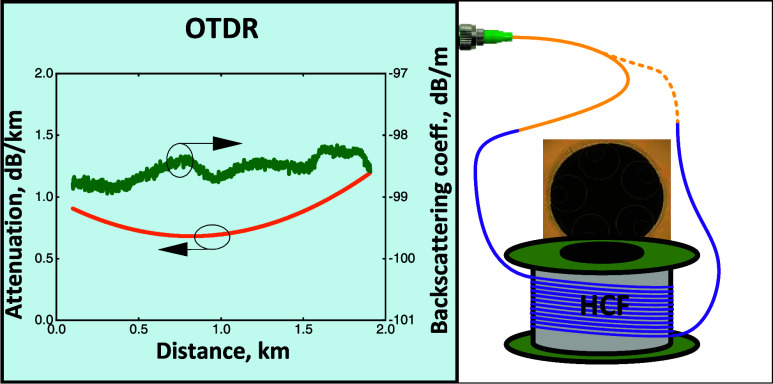

Optical time domain reflectometry (OTDR) is a key technique to characterize fabricated and installed optical fibers. It is also widely used in distributed sensing. OTDR of emerging hollow-core fibers (HCFs) has been demonstrated only very recently, being almost 30 dB weaker than that in the glass-core optical fibers. However, it has been challenging to extract useful data from the OTDR traces of HCFs, as the longitudinal variation in the fiber’s geometry, notably the core size or the longitudinal variations of the air pressure within the core, results in commensurate changes of the backscattering strength. This is, however, necessary for continuous improvement of HCF fabrication and subsequent improvement in their performance such as minimum achievable loss, potentially enabling the use of HCF in a significantly broader range of applications than used today. Here, we demonstrate, for the first time, that the distributed loss and backscattering coefficient in antiresonant HCFs can be separated, obtaining key data about fiber distributed loss and uniformity. This is enabled by using OTDR traces obtained from both ends of the HCF.

## Introduction

For today’s conventional optical fibers such as standard single-mode fiber (SMF), optical time domain reflectometry (OTDR) is routinely used both in manufacturing and in installation environments. It provides comprehensive information such as attenuation, position of faults, high loss (e.g., due to excessive bending during installation, dirty connector, or a bad splice), and position of connectors,^1,2^ all by accessing only one end of the fiber and providing results within minutes.

When interpreting OTDR traces, attenuation is typically extracted from the slope of the measured distributed signal, which originates from Rayleigh backscattering in the fiber glass core that is constant along the fiber length in today’s SMFs.

OTDR has also been used in emerging low-loss hollow-core fibers (HCFs) such as the (double) nested antiresonant nodeless fibers (NANFs/DNANFs).^3,4^ Extraction of attenuation using OTDR presented here has been instrumental in the latest step to finesse their fabrication, resulting in the recent lowest attenuation reported of any optical fiber ever made at <0.11 dB/km, with preliminary results shown in ref (5). In these HCFs, the dominant backscattering mechanism is usually the backscattering from the air/gas in the core region.^6^ Subsequently, the strength of the backscattering signal (characterized by the backscattering coefficient B) can vary due to air pressure/composition within the core along the HCF length, which can change with time as the air can move within the core region.^7,8^ Further, it has been predicted that the backscattering coefficient also changes with the core size,^6^ which varies at the micrometer level in today’s low-loss HCFs.^9^ This has made evaluation of the distributed HCF attenuation coefficient challenging, as the OTDR signal depends simultaneously on the backscattering coefficient and attenuation, both expected to vary along the fiber length.

Similar phenomena were observed in very early SMFs in which the backscattering coefficient also changed along the fiber length, not allowing the attenuation coefficient to be directly extracted from the OTDR traces. It has been, however, shown that by measuring the OTDR trace from both fiber ends, it is possible to extract the distributed attenuation coefficient and the backscattering coefficient.^10^ Here, we adapt this two-way OTDR measurement technique to measure the distributed backscattering coefficient and attenuation coefficient along the low-loss antiresonant HCFs. It has already been instrumental in the developing world’s lowest-attenuation HCFs.^5^ Besides introducing this technique, we also show here an error analysis of this powerful method, considering measurement errors, and found that the measured value of the backscattering coefficient B agrees with predictions made via simulations within the measurement error. Accumulated loss extracted from the OTDR agrees with cutback measurements, further confirming the accuracy of the demonstrated method. The level of achieved accuracy has already proven to be instrumental in further developments of low-loss HCF, with achieved attenuation below that achievable in today’s glass-core fibers. Further attenuation reduction is expected with the help of here-presented technique. This is promising to not only revolutionize the capability of science and technology fields that are already using fiber optics (optical communications,^11^ high-power fiber laser,^12^ or high-power laser delivery,^13^ etc.) but also empower new fields where current optical fibers have limited use due to their impairments such as quantum communications and computing.^14^

## Principle of Two-Way OTDR

**Figure 1:**
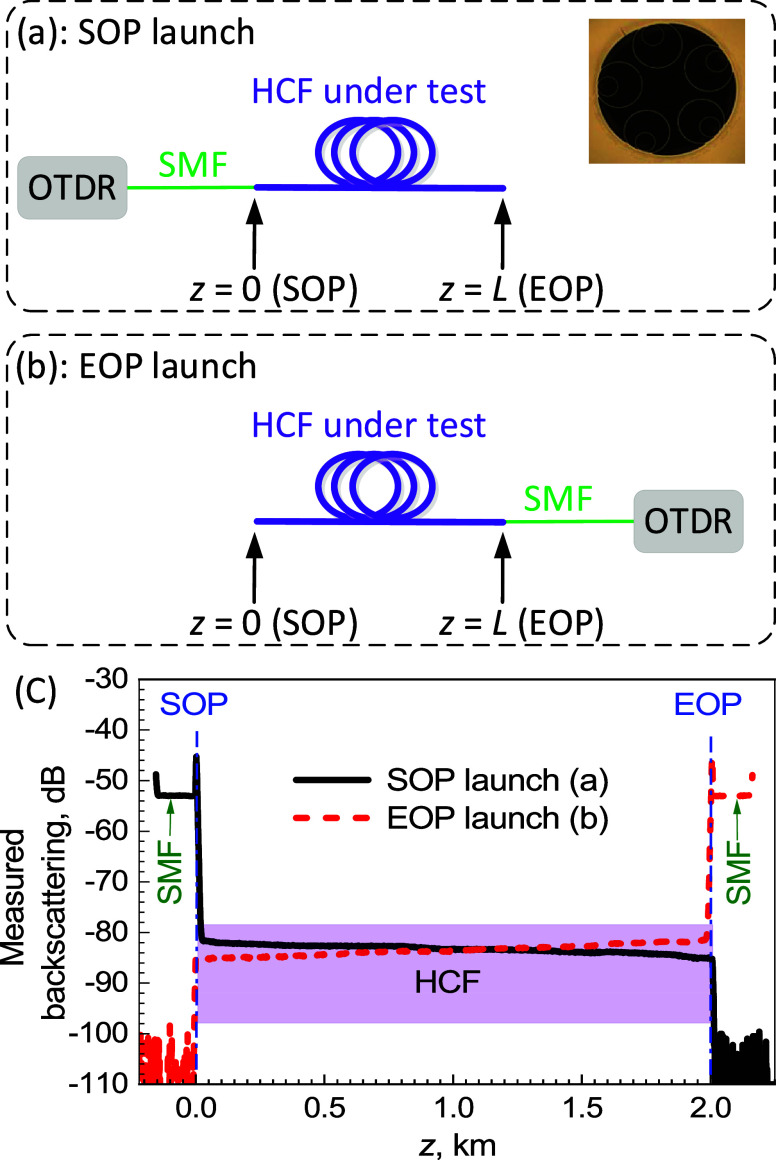
OTDR with an SMF pigtail connected into the HCF under test via (a) SOP end (Inset: Cross section of HCF-1 (NANF type)) and (b) EOP end. (c) Measured traces on the HCF-1 sample.

We consider an HCF with length *L* and mark its beginning (*z* = 0) as “start of pull” (SOP) and its end (*z* = *L*) as “end of pull” (EOP), Figure 1. Subsequently, we consider measuring two OTDR traces when launching OTDR pulses into the SOP (Figure 1a) and EOP (Figure 1b) ends, respectively. Examples of measured traces using a 2.0 km long HCF sample with NANF geometry (end-face shown as inset in Figure 1a) are shown in Figure 1c. We refer to this sample as HCF-1 and give further details on it in the Experimental Results Section.

In Figure 1c, at first glance, we see both measured traces decreasing along the propagation direction due to the HCF under test attenuation, similar to OTDR traces of an SMF fiber. More detailed observation, however, shows discrepancies. For example, around *z* = 1.8 km, the EOP launch trace (red, dashed) is almost flat, suggesting very small HCF attenuation, while the SOP launch trace (black, solid) decreases with propagation distance significantly, suggesting high HCF attenuation. As we show later, this is due to the variation in the backscattering coefficient B that, in this example, decreases with *z* around *z* = 1.8 km. This example suggests that the backscattering coefficient and attenuation variation can be separated when analyzing SOP and EOP launch traces, which is confirmed by the theoretical analysis shown in the following section.

### Analysis of Two-Way OTDR

#### Derivations

The backscattered power received from the SOP and EOP launch traces can be expressed as
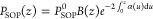
1
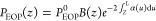
2Here, *P*^0^ is the launched power into the HCF from SOP (subscript “SOP”) and EOP (subscript “EOP”) ends, respectively. *B*(*z*) and α(*z*) are backscattering and attenuation coefficients at point *z* along the HCF.

By applying natural logarithm and summing eqs 1 and 2, we obtain
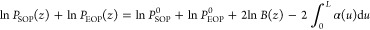
3

By defining *A* = ∫_0_^*L*^α(*u*)d*u* as the total HCF loss, we obtain the backscattering coefficient from eq 3 as
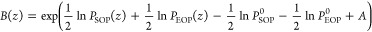
4

When all variables are converted into d*B*, we obtain
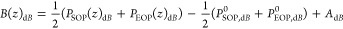
5

Subsequently, accumulated loss is obtained from eqs 1 and 4:
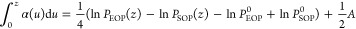
6

In d*B*, it is then:
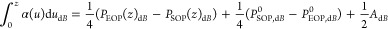
7

The HCF attenuation coefficient at point *z* is then obtained from the derivate of eq 7:
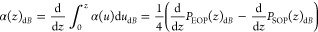
8

The backscattering coefficient *B*(*z*)_d*B*_ is then evaluated using eq 5, where the total HCF loss *A*_d*B*_ is evaluated from eq 7 by putting *z* = *L*.

#### Practical Considerations and Calibration

The first practical consideration regards the differentiation of data measured experimentally to obtain an attenuation coefficient from eq 8, as differentiation of noisy experimental data usually produces large noise variation. We will use the approach adopted in the analysis of the SMF OTDR traces in which the accumulated loss of ∫_0_^*z*^α(*u*)d*u*_d*B*_ given in eq 7 is first fitted with a polynomial, and it is subsequently differentiated to obtain the attenuation coefficient α(*z*)_d*B*_.

**Figure 2:**
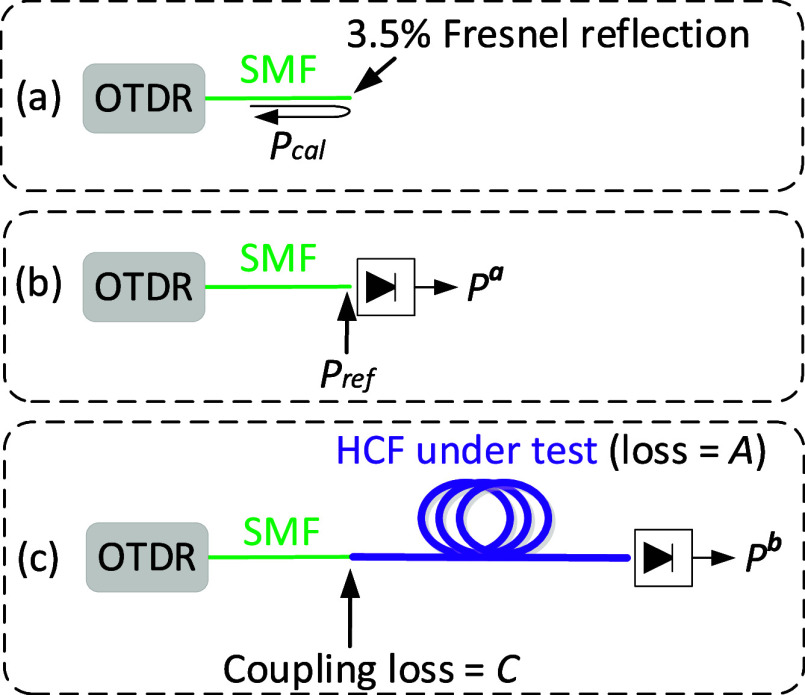
Setup for calibrating the OTDR instrument (a) and to measure powers of *P*^*a*^ (b), *P*^*b*^ (c) used to evaluate quantities of *P*_SOP_^0^ or *P*_EOP_^0^.

To find *P*_SOP_(*z*)_d*B*_ and *P*_EOP_(*z*)_d*B*_, we need to calibrate our OTDR, for which we use a 3.5% Fresnel back-reflection from the flat-cleaved SMF, Figure 2a. Subsequently, we evaluate all quantities relative to the powers expected inside the SMF at its output: this point is shown in Figure 2b as *P*_ref_. The details of calibration are shown later.

Further, we need to derive the backscattering coefficient (eq 5) from the quantities that can be measured experimentally rather than the *P*_SOP,d*B*_^0^, *P*_EOP,d*B*_^0^, and *A*_d*B*_ used in eq 5. We consider experimentally measurable quantities of power at the launching SMF output, *P*^*a*^ (Figure 2b), and at the output of the HCF under test (Figure 2c), *P*^*b*^. Below, we show derivation for SOP launch, as EOP launch can be treated identically. First, the difference between the power measured after and before the HCF under test is due to the HCF loss *A*_d*B*_ and coupling loss *C*_d*B*_ (Figure 2c):
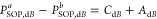
9

Further, light propagating in the SMF tail experiences two times coupling loss (2*C*_d*B*_) when backscattered plus Fresnel loss *F*_d*B*_ (*F*_d*B*_ = 0.15 dB) when entering SMF into the HCF:
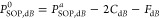
10

Note that the Fresnel loss for the SMF-HCF direction is already accounted for, as *P*^*a*^ is measured after light experiences this Fresnel loss in the forward direction (*P*_ref,d*B*_ = *P*_d*B*_^*a*^ + *F*_d*B*_). Equations 9 and 10 lead to
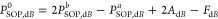
11and similarly for the opposite direction:
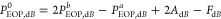
12

We now can evaluate the total HCF loss *A*_d*B*_ by using eq 7 and putting *z* = *L*:
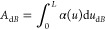
13
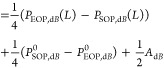
giving:
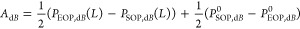
14

Using eqs 11 and 12 to replace *P*_SOP,d*B*_^0^ and *P*_EOP,d*B*_^0^ in eq 14, we get:
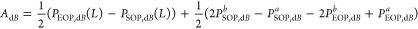
15

We then put eqs 11, 12, and 15 into eq 5 to obtain the backscattering coefficient from measured quantities:
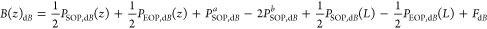
16

The accumulated loss *A*(*z*)_d*B*_ can then be obtained from eq 7 using eqs 11, 12, and 15 as
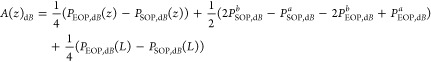
17

In regard to the attenuation coefficient α(*z*)_d*B*_, eq 8, it does not require *P*_SOP,d*B*_^0^, *P*_EOP,d*B*_^0^, or *A*_d*B*_, and even calibration of the OTDR is not necessary.

Now, having all of the parameters of interest related directly to the measurable quantities, we can estimate the accuracy with which we can evaluate them.

### Error Analysis

**Figure 3:**
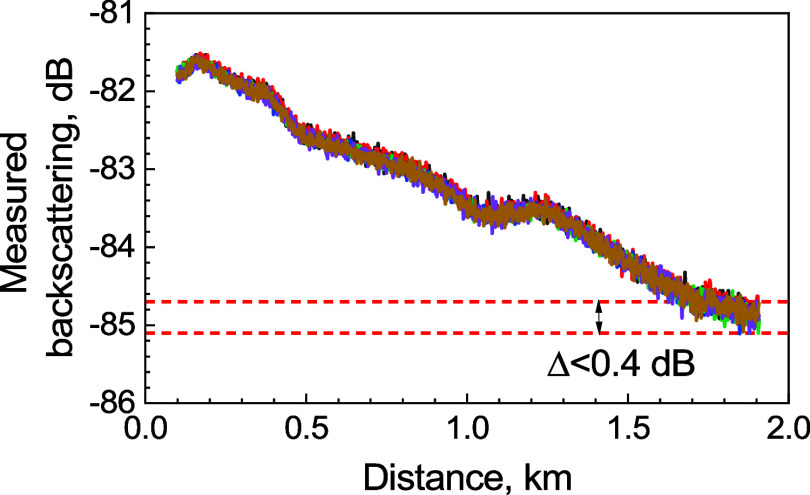
Six backscattering traces measured on the HCF-1 sample.

First, we estimate measurement errors. To evaluate the error of the OTDR measurements of *P*_SOP,d*B*_ (*z*) and *P*_EOP,d*B*_ (*z*), we performed repeated measurements of the HCF-1 sample, Figure 3. The peak-to-peak variation between the traces at any given distance *z* is below 0.4 dB. Thus, we estimate uncertainty in measuring *P*_SOP,d*B*_ (*z*) and *P*_EOP,d*B*_ (*z*) as ±0.2 dB. The error of the measured powers of *P*_SOP,d*B*_^*a*^, *P*_EOP,d*B*_^*a*^, *P*_SOP,d*B*_^b^, and *P*_EOP,d*B*_^b^ is given by the accuracy of the used power meter, which is given by the manufacturer as ±5%, corresponding to ±0.2 dB.

With the above estimation of the measurement errors, the error in evaluated *B*(*z*)_d*B*_ given by eq 16, calculated as peak-to-peak errors (all errors summed up), is as follows:
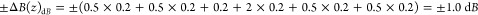
18

In regard to the error in α(*z*)_d*B*_, it does not contain directly any of the measured powers (eq 8), and thus, their error should not contribute to the error in α(*z*)_d*B*_. It also does not contain the OTDR data directly, but only their derivatives (eq 8), which we will smooth out by performing the earlier-mentioned polynomial fit. Thus, we expect that the error in α(*z*)_d*B*_ will depend dominantly on the quality/accuracy of this polynomial fit. Other parasitic effects may also play a role, e.g., when measuring an HCF with subatmospheric air pressure inside, air will be getting in during the measurement. This could be reduced by measuring OTDR from both ends simultaneously using two instruments. The most important conclusion, however, is that the accuracy of α(*z*)_d*B*_ is not influenced by the accuracy of any measured powers, and being so, it can be significantly more accurate than the accuracy of the power measurements, making this method potentially very accurate.

#### OTDR System

**Figure 4:**
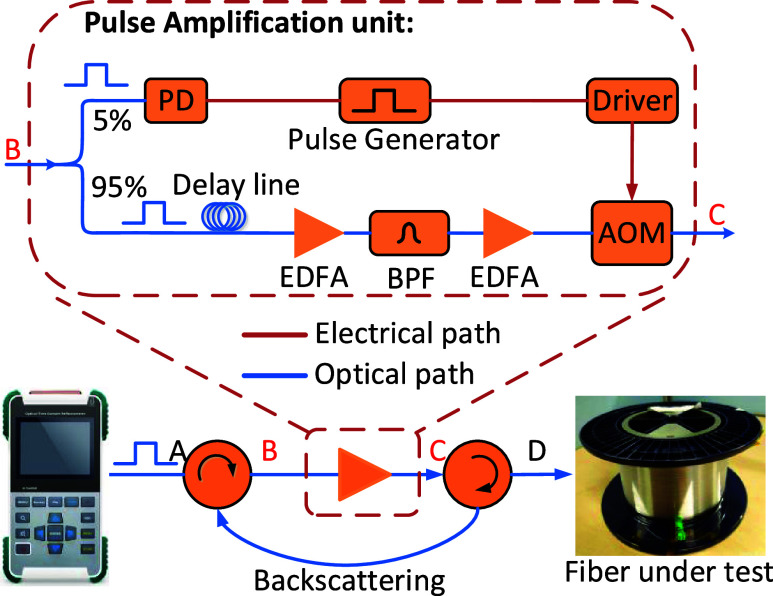
(a) Setup of the used highly sensitive OTDR system and (b) detail of the implemented pulse amplification unit. PD: photodetector; AOM: acousto-optic modulator; BPF: bandpass filter; EDFA: erbium doped fiber amplifier.

The custom OTDR system we built for measuring the backscattering in HCFs is shown in Figure 4, and it was first demonstrated in ref (3). To enhance the overall sensitivity of the OTDR (FOTR-203 from FS.com), the OTDR pulses were amplified before they were injected into the HCF under test. This was achieved by employing a “pulse amplification” unit with two inline optical circulators, which ensure that the backscattered signal is directed back into the OTDR instrument. Detailed discussion is found in ref (3).

Using this OTDR system, the launched pulses were amplified by more than 28 dB, enabling measurement of the backscattering signal in HCFs with a spatial resolution of 1.5 m (10 ns pulses), as demonstrated in ref (3).

#### OTDR Calibration

**Figure 5:**
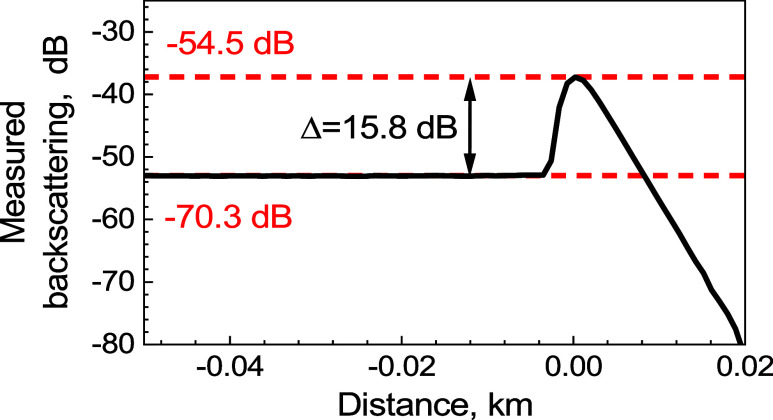
Measured backscattering from the SMF pigtail and Fresnel reflection (attenuated by 40 dB) at its end.

We calibrated the OTDR following the procedure shown in Figure 2a, where we used 10 ns flat-top pulses. The measured OTDR trace is shown in Figure 5. As the 3.5% reflection (corresponding to −14.5 dB) is very strong and beyond the dynamic range of the OTDR detector, we placed a variable optical attenuator at the output and set it to 20.0 dB by comparing the output power with and without the attenuator. This reduced the back-reflected light by 40.0 dB, as the light passes through the attenuator in both directions. The detected peak from the 3.5% back-reflecting end-facet should thus be −14.5–40.0 =–54.5 dB below light launched into the fiber, as schematically shown in red in Figure 5. As we measured the back-reflected peak of −37.2 dB (Figure 5), we needed to subtract 17.3 dB from the measurement of −37.2 dB to obtain a correct value of −54.5 dB. 17.3 dB is thus our calibration constant of the OTDR relative trace.

In Figure 5, we see that the backscattering from the SMF is 15.8 dB below that of the 3.5% end-facet. As the used 10 ns pulse length corresponds to the OTDR signal received from 1 m of SMF, we get the backscattering coefficient of the SMF after our calibration as −54.5–15.8 =–70.3 dB/m, as shown in red in Figure 5. This value is consistent with SMFs,^15^ providing us with the verification of the calibration as well as providing us with the accurate value of the backscattering coefficient of our SMF.

**Figure 6:**
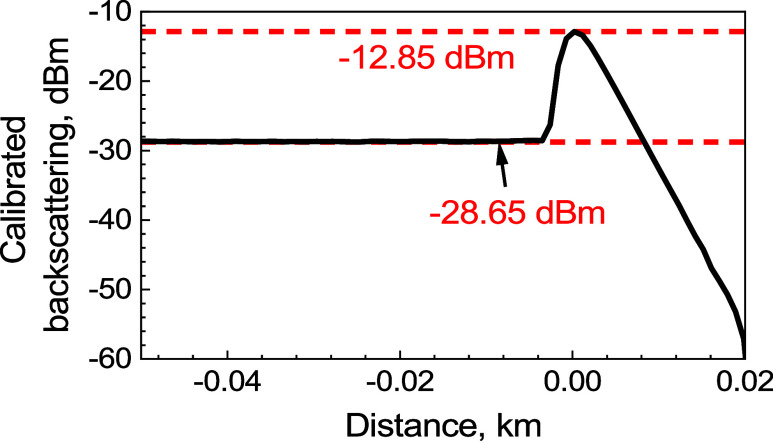
Calibration of the measured backscattering from the SMF pigtail and Fresnel reflection (attenuated by 40 dB) at its end.

The above calibration gives us relative powers (in dB); however, we also require absolute powers (in dBm) for the evaluation of *B*(*z*)_d*B*_ and *A*(*z*)_d*B*_, eqs 16 and 17. To obtain it, we measured the pulse power of *P*_ref_ = *P*_*a*_ + 0.15 = 41.65 dBm. Thus, the Fresnel reflection that is expected at −54.5 dB below a power of 41.65 dBm is −12.85 dBm, which gives us absolute calibration of the OTDR traces. The calibrated measured trace from Figure 5 in absolute power (dBm) is shown in Figure 6.

## Experimental Results

### Used Samples

To demonstrate the capability and accuracy of the developed technique, we characterized three HCF samples, which we mark as HCF-1, HCF-2, and HCF-3, as shown in Table 1. The core sizes we refer to are those measured at one of the HCF sample end-facets analyzing scanning electron micrograph (SEM) images.

**Table 1:** Parameters of the Characterized HCFs

sample	HCF-1	HCF-2	HCF-3
type	NANF	NANF	DNANF
core size, μm	34.7	31.0	26.7
length, km	2.0	0.68	6.5
air pressure, atm	1	1	0.25^a^

a Close-to “as-drawn”, which has subatmospheric air pressure. Value estimated based on the backscattering level.^6^

The HCF-2 sample that has a relatively large bending loss was initially measured on a standard fiber bobbin (a diameter of 16 cm) and subsequently rewound and remeasured on a large bobbin (a diameter of 32 cm).

### Measured OTDR Traces (Calibrated)

**Figure 7:**
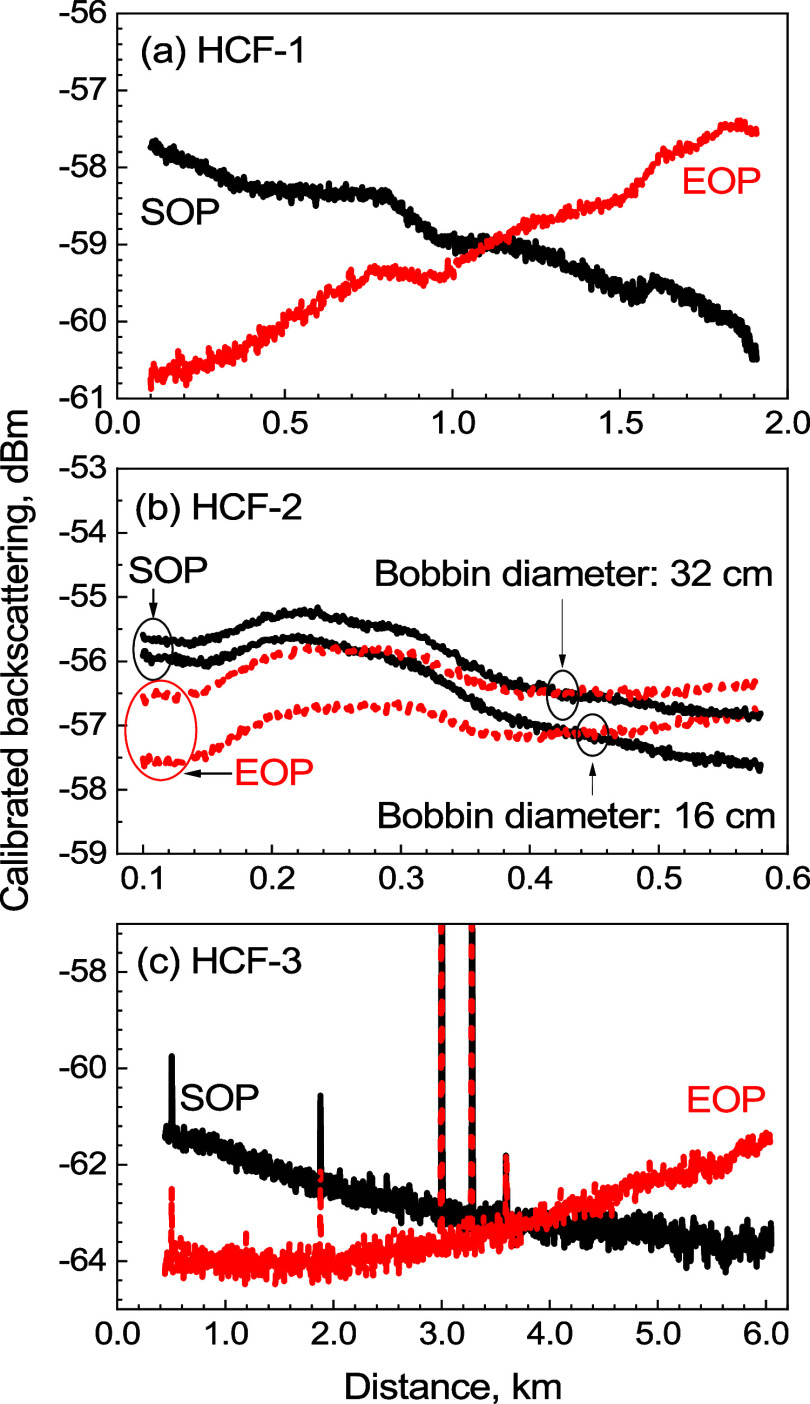
Calibrated backscattering traces when launching light from the SOP and EOP for (a) HCF-1, (b) HCF-2 on a small (solid) and large (dashed) bobbin, and (c) HCF-3.

We measured the backscattering in HCF-1, HCF-2, and HCF-3, and calibrated the traces as described earlier, with the result shown in Figure 7. We removed data at both ends of the samples to avoid the influence from the dead zone of the instrument.

### Attenuation Coefficient

**Figure 8:**
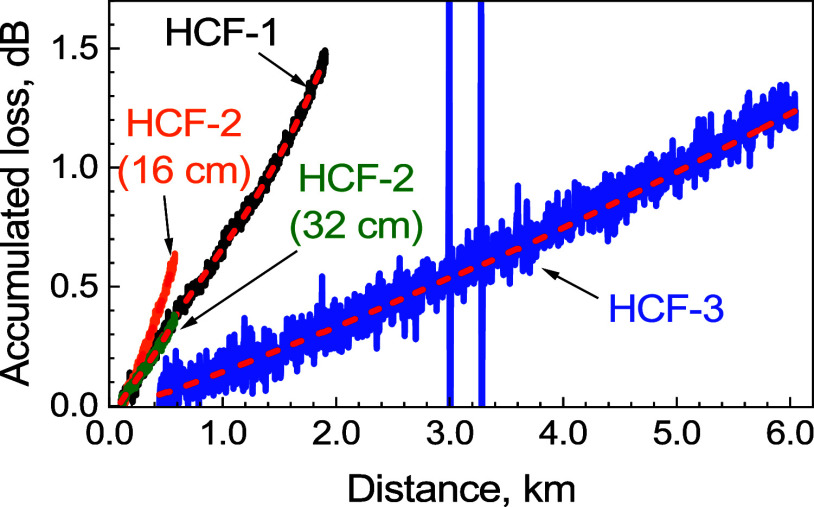
Accumulated loss for all three HCF samples and their third-order polynomial fits (red dashed lines).

**Figure 9:**
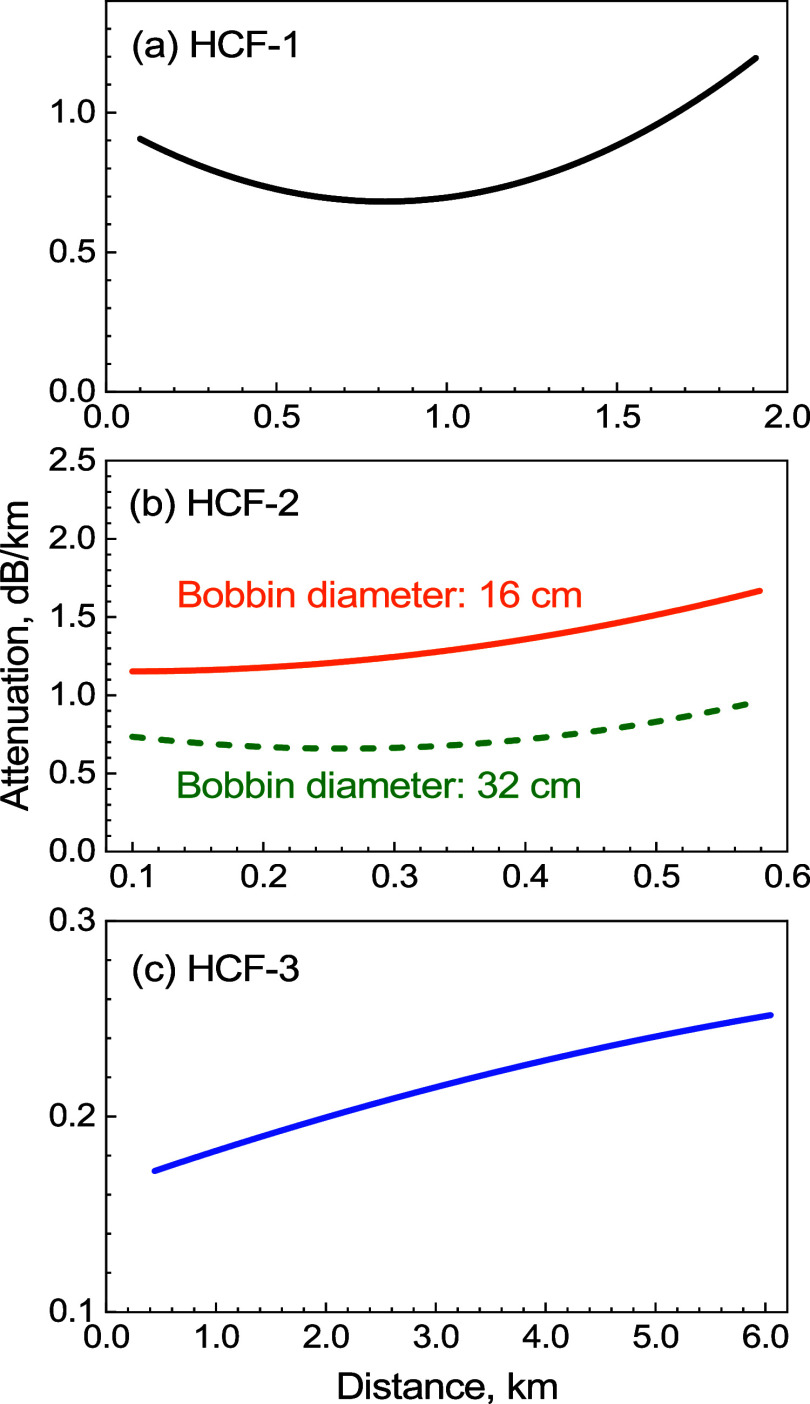
Attenuation coefficients for (a) HCF-1, (b) HCF-2, and (c) HCF-3.

We calculated the accumulated loss as described earlier when discussing eqs 17 and 8. Results are shown in Figure 8 for all three HCF samples. To obtain attenuation coefficients, we first fitted this accumulated loss data with a polynomial function and subsequently differentiated it. All accumulated loss traces were fitted by the first-, second-, ··· order polynomials, and the coefficient of determination (*R*^2^) was obtained for each fit. Subsequently, we used a fit with the polynomial order from which *R*^2^ did not change within four digits. For example, for HCF-1, the *R*^2^ values were 0.9932, 0.9956, 0.9972, and 0.9972 for the first-, second-, third-, and fourth-order polynomial fits, respectively. Subsequently, we chose the third order, as *R*^2^ did not change between the third- and fourth-order polynomials. We repeated this analysis for HCF-2 and HCF-3 samples, which suggested using a cubic fit for all three measured samples, as shown in Figure 8. The attenuation coefficient profiles (derivatives of the fitted curves) are then shown in Figure 9a–c.

### Backscattering Coefficient

**Figure 10:**
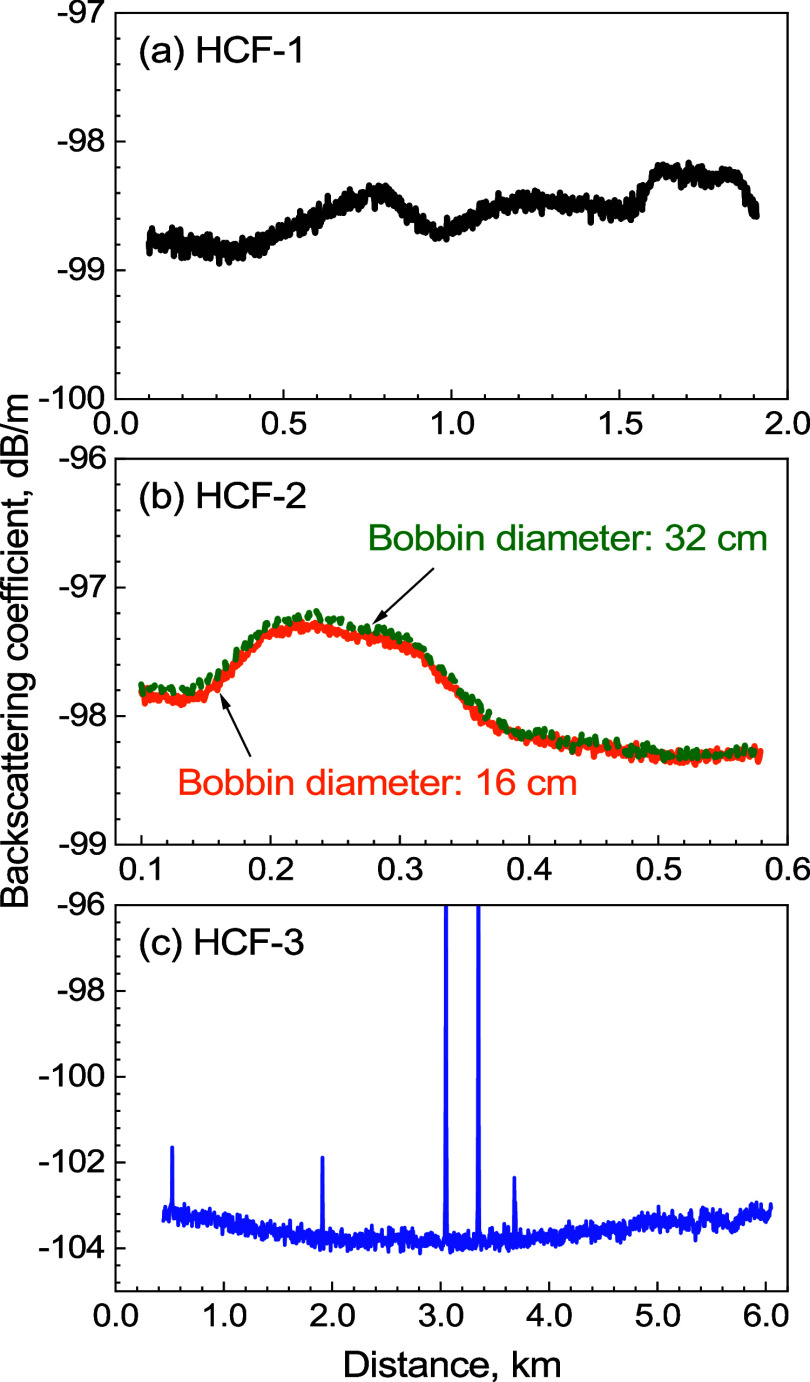
Backscattering coefficients for (a) HCF-1, (b) HCF-2, and (c) HCF-3.

The backscattering coefficients of the three samples are then calculated by using eq 16 and are shown in Figure 10. A variation of about ±0.5 dB along the length is observed for all three samples. This can be caused by the core size variations^6^ or air pressure variations inside the core.^7^ In Figure 10b, the backscattering coefficient for the HCF-2 sample spooled on different size bobbins (having different attenuation coefficients) shows a remarkably similar value, showing negligible cross-sensitivity of the attenuation changes on the evaluation of the backscattering coefficient.

## Discussion

We compare the attenuation coefficients obtained from the two-way OTDR analysis to the average attenuation coefficient obtained via the cutback method (Table 2). Results for HCF-1 and HCF-3 show good agreement between the two techniques. For HCF-2, the fiber has been rewound between the measurements to a smaller and back to a larger spool, which may have slightly altered the attenuation, possibly explaining the OTDR result to be slightly higher than expected from the cutback method.

**Table 2:** Attenuation Coefficient Extracted from the OTDR Measurement and Its Comparison to the Average Attenuation Coefficient Measured by the Cutback Method

sample	attenuation coefficient variation along the length, OTDR, dB/km	average attenuation coefficient from cutback, dB/km
HCF-1	0.69–1.20	0.85 ± 0.03
HCF-2	0.66–0.96 (32 cm)	0.6 ± 0.1
HCF-3	0.17–0.25	0.21 ± 0.01

**Figure 11:**
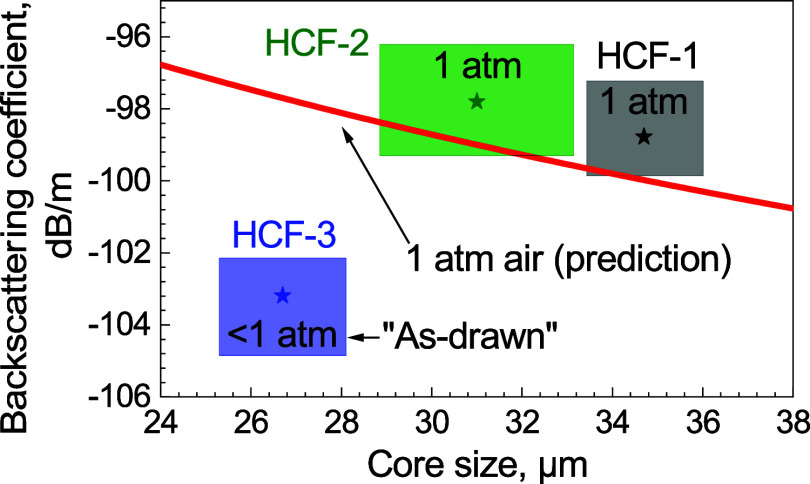
Comparison between the extracted backscattering coefficient of three samples and the predicted value in ref (6).

We also compare the obtained backscattering coefficients with the prediction,^6^ as shown in Figure 11 and Table 3. The asterisks shown in Figure 11 are the backscattering coefficient level at the SOP sides. The uncertainties are marked in color regions, which combine the variation of the backscattering coefficient shown in Figure 10 along the HCF sample lengths and the error of ±1 dB that was analyzed earlier. The core size range is estimated based on the relationship between backscattering coefficient variation and core size predicted in ref (6) (here, we use 0.25 μm/dB for the calculation). For HCF-1 and HCF-2, both at atmospheric-pressure air-filled, they agree with the prediction. The HCF-3 shows a lower backscattering coefficient; however, we know that this sample was sealed after the fiber draw, and thus, the air pressure inside it is expected to be below 1 atm, qualitatively in line with our measurement. As its backscattering coefficient is about 6 dB lower than expected, we estimate air pressure inside this sample of 0.25 atm, which is consistent with earlier reports showing as-drawn HCFs to have air pressure as low as 0.2 atm.^16^

**Table 3:** Backscattering Coefficient Extracted from the OTDR Measurement and Its Comparison to the Prediction in Reference (6)

	backscattering coefficient variation along the length + uncertainty, OTDR, dB/m	backscattering coefficient from theory (1 atm) dB/m
HCF-1	–99.9 to −97.2	–100.3 to −99.6
HCF-2	–99.3 to −96.2 (32 cm)	–99.6 to −98.4
HCF-3	–104.9 to −102.2^a^	–98.1 to −97.2

a HCF-3 had subatmospheric pressure, as it has been as drawn.

## Conclusions

We have demonstrated that HCF’s distributed attenuation and backscattering coefficients can be accurately obtained from OTDR traces by using two-way OTDR measurement. By analyzing in detail three low-loss antiresonant HCFs, we demonstrated the capability of the method used in separating loss from backscattering coefficient variation. In terms of loss, we obtained good agreement between the loss extracted via two-way OTDR and the cutback method. A slight variation of the attenuation coefficient along the length (e.g., between 0.17 and 0.25 dB/km over 6.5 km of HCF-3 sample length) can be useful in developing low-loss HCFs. In terms of the backscattering coefficient, we achieved a close agreement with the values expected from simulations.^6^ The observed slight variation along the length can be attributed to air pressure variations or HCF core size variations,^6^ suggesting that this method can give direct insight into the longitudinal uniformity of the drawn HCFs, providing valuable feedback to their manufacturing as well as quality. We believe the demonstrated method will be a powerful tool in further development of HCFs, approaching their fundamental limits and thus providing next-generation optical fibers to a multitude of applications. Examples of these applications are not only optical communications and manufacturing but also fields of long-distance distributed sensing or unamplified transmission of qubits over large distances.
